# Assessment of plant species diversity based on hyperspectral indices at a fine scale

**DOI:** 10.1038/s41598-018-23136-5

**Published:** 2018-03-19

**Authors:** Yu Peng, Min Fan, Jingyi Song, Tiantian Cui, Rui Li

**Affiliations:** College of Life & Environmental Sciences, Minzu University of China, Haidian District, Beijing 100081 China

## Abstract

Fast and nondestructive approaches of measuring plant species diversity have been a subject of excessive scientific curiosity and disquiet to environmentalists and field ecologists worldwide. In this study, we measured the hyperspectral reflectances and plant species diversity indices at a fine scale (0.8 meter) in central Hunshandak Sandland of Inner Mongolia, China. The first-order derivative value (FD) at each waveband and 37 hyperspectral indices were used to assess plant species diversity. Results demonstrated that the stepwise linear regression of FD can accurately estimate the Simpson (R^2^ = 0.83), Pielou (R^2^ = 0.87) and Shannon-Wiener index (R^2^ = 0.88). Stepwise linear regression of FD (R^2^ = 0.81, R^2^ = 0.82) and spectral vegetation indices (R^2^ = 0.51, R^2^ = 0.58) significantly predicted the Margalef and Gleason index. It was proposed that the Simpson, Pielou and Shannon-Wiener indices, which are widely used as plant species diversity indicators, can be precisely estimated through hyperspectral indices at a fine scale. This research promotes the development of methods for assessment of plant diversity using hyperspectral data.

## Introduction

The fast and nondestructive estimation of plant species diversity has received increasingly more attention from ecologists in recent decades^1,2^. Remote sensing facts offer composite data which can sense the features of an item and mirror its real standing, agreeing on a considerable decrease in field survey costs and labor. Such methods display great potential for estimating plant diversity^3^. Spectral heterogeneity among plant species is related to the variation in plant species and thus can be considered as one method to specify plant species diversity^3,4^. Theoretically, data obtained by remote sensing of adequate spectral resolution could indicate plant species diversity, but the identification of the appropriate spectral bands is challenging.

Near infrared^4^, middle infrared^5^ and thermal infrared bands^6,7^ have been strongly suggested for species diversity discrimination. Their combinations were also verified robust indicators of plant diversity. The combination as Normalized Difference Vegetation Index (NDVI) used to estimate species richness^8–10^ and Shannon and Simpson diversity indices^11,12^, Enhanced Vegetation Index (EVI) can minimize atmospheric noise and soil background and improve the estimation of plant diversity in the case of dense canopy^13,14^. Other combinations as Infra Red Index (IRI), Middle Infra Red Index (MIRI), Atmosphere Resistance Vegetation Index (ARVI) and Soil Adjusted Vegetation Index (SAVI) were also found to closely relate to plant species diversity^7,10,13,15^.

Numerous statistical methods have also been used to reform the excellence in plant diversity. Linear regression^11^, hierarchical agglomerative cluster^16^, standard deviations^17^ as well as the first^17,18^ and second^18^ order derivatives of reflectance values were all used for diversity material extraction and validated a good fit between the outcomes and plant diversity indices. Even though previous researchers have discovered the near connection between plant diversity and spectral indices, the coarse spectral and spatial resolutions have limited the estimation accuracy^19,20^. Higher spectral variation is closely related to higher environmental heterogeneity, thus indicated the possibility of higher species diversity^21^. Hyperspectral data have hundreds of wavebands and the highest spectral resolution. They can depict greater detail in spectral heterogeneity as well as image plant diversity.

Plant species diversity was successfully predicted by the hyperspectral indices (with an error of ca. 20%) within a 4 m^2^ scale in dry grazed grasslands in Sweden^20^, as well as in a temperate forest in Germany (R^2^ = 0.76)^22^. The combination with hyperspectral data (4 meter resolution), the WorldView-2 imageries can statistically significantly improve species classification accuracy (79% ± 1.8) compared to the WorldView-2 data alone (77% ± 3.1)^23^. However, the estimating accuracy is also affected by the spatial resolution of hyperspectral data. The hyperspectral data from airborne sources are made at a coarser scale (4–30 meters) and tend to provide an average for several aspects—such as healthy and diseased leaves, stems, and even the shadows and orientation of the woody plant shoot rather than canopy alone. These data are largely affected by the illumination conditions of species measuring and canopy reflectance, hence the biodiversity estimation^24^. Additionally, hyperspectral imagery is expensive and not readily available. The high cost, atmospheric noise, and coarse resolution have considerably limited the wide direct application of hyperspectral data from airborne imagery.

Associate to the aloft borders, the hyperspectral facts at a good scale (less than 1 meter) can gather the detailed reflectance data by less inspiration from background environment and atmosphere by the benefit from the fine scale, may be regarded as a desirable source to extract plant diversity information. However, to the best of our best knowledge, few studies reported, the direct relationship between hyperspectral data and plant species diversity at a fine scale. We assume that 1) hyperspectral data at a fine scale can accurately estimate plant diversity, and 2) spectral indices based on sensitive bands can get more desirable results than based on empirical bands. In order to test these hypotheses, we conducted a field study in the Hunshandak Sandland, Northern China, collected field-survey vegetation parameters and hyperspectral data for the pant diversity indices, exploring the potential of hyperspectral data in estimating plant diversity.

## Methods

### Study area

**Figure 1:**
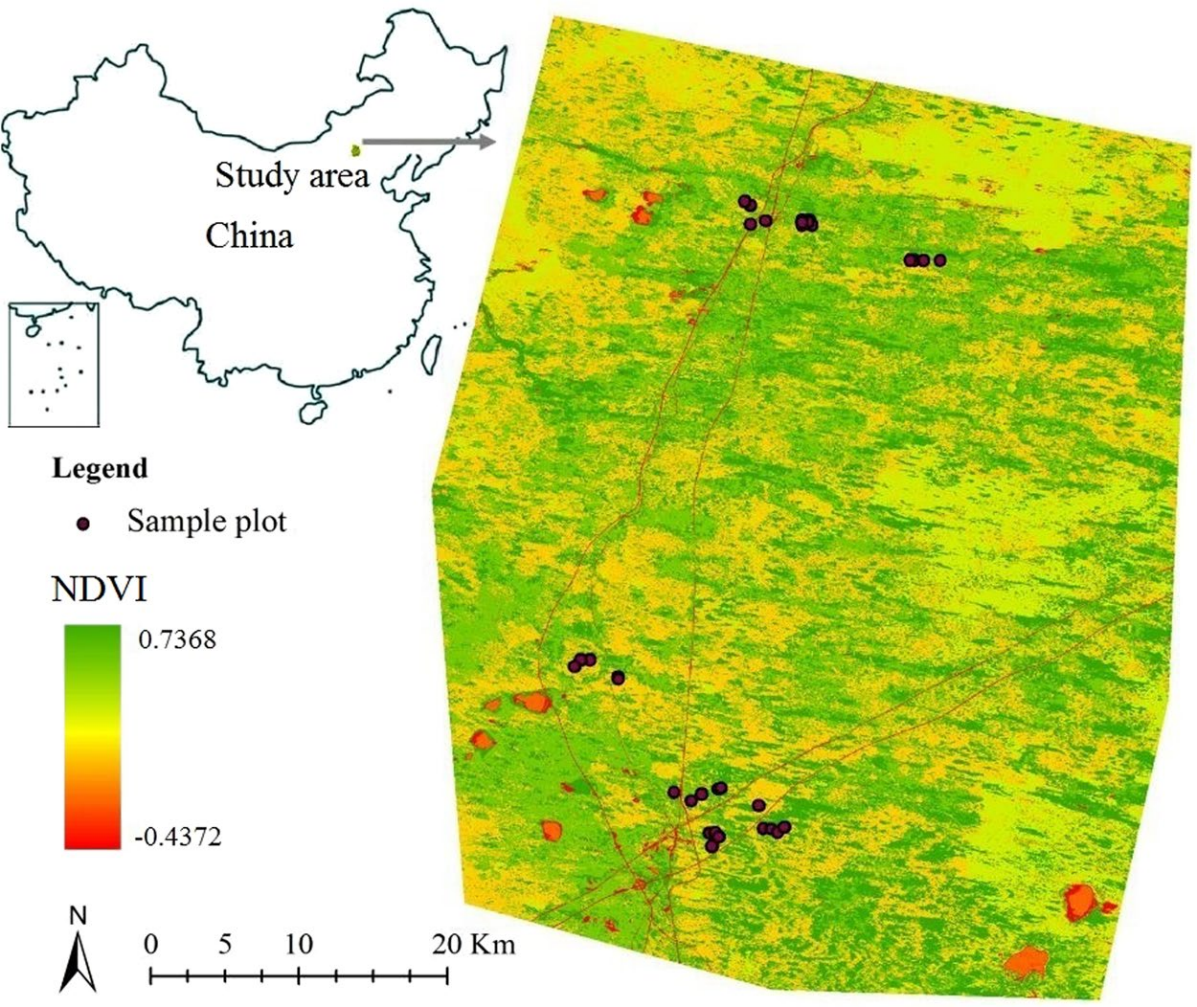
Location of study area: the central Hunshandak Sandland in China, and samples location. Map created using ArcGIS 10.2 software (http://www.esri.com) by the first author.

The study was conducted in temperate grassland of Hunshandak Sandland (41°46′-43°69′N, 114°55′-116°38′E), Inner Mongolia, Northern China (Fig. 1). The prevailing climate is of temperate semi-arid type with an annual mean temperature of 1.7 °C. The diurnal minimum and maximum monthly temperature is −18.3 °C and 18.7 °C, respectively. Hunshandak Sandland receives an annual precipitation of 250–350 mm, 80–90% of which falls between May and September. Semi-natural grasslands in Hunshandak Sandland are associated with high levels of species-rich habitats at a fine scale, where shelter most plant species, hence prevent the movement of sandy dunes and desertification process^25^. Operative scheme for the rapid assessment of fine-scale (0.25–1 meter) plant species diversity are therefore needed for monitoring of ecological status in species-rich habitats. The landscape of Hunshandak Sandland possesses a unique pattern consisting of fixed sandy dunes, semi-fixed sandy dunes, moving dunes, and lowland where relatively rich in plant diversity. These fine-habitats with rich plant diversity and the nearly flat landform mark it a perfect region to discover the relationship between hyperspectral data and plant species diversity at a fine-scale.

### Plant diversity survey and analysis

A total of 24 plots, each measuring 10 m by 10 m, randomly distributed throughout the northern and southern central Hunshandak Sandland were chosen for the survey. The geographical position of centre in each sampling plot was acquired using a high-precision GPS (accurate to within a metre). Within each plot, five subplots (circles with diameter of 0.8 meter each) were marked off, each measuring a diameter of 0.8 m and originating from different corner and centre of the main plot. The abundance, cover and height of each plant species and habitat categories (fixed sandy dunes, semi-fixed sandy dunes, mobile sandy dunes, lowland, water, and construction land) were recorded in July of 2016. The number of individuals was recorded for species whose stems were either fully or partially within the subplot. The clonal species were accounted as separate individuals if stems or culms were larger than 20 cm from others belonging to the same species. Canopy cover was visually estimated for all species with canopy cover within the subplot. Reliability of visual approximation was preserved by the assessment being done by the same person all over all plots. Completely, the vegetation parameters of 120 subplots were acquired.

Based on the collected data on the abundance of plant species, we calculated five widely-used indexes of biodiversity, namely the Shannon–Wiener index, the Simpson species evenness index, the Pielou index, the Margalef index and the Gleason index^26^, using the following formulas for each circle.

To estimate alpha diversity, we calculated the Shannon–Wiener index (*H*) (1).

1 $$H=-\sum _{i=1}^{s}\frac{{n}_{i}}{N}\,\mathrm{ln}\,\frac{{n}_{i}}{N}$$

where *H* is the diversity in a circle of *S* species, *n*_*i*_ is the number of individuals of the *i*th species, *N* is the total number of individuals of all the species, and ln is the natural logarithm. The higher value of *H* means higher species richness and also signifying that different species in the quadrat or a community are nearly equally abundant. Shannon index is one of the most widely used measures of diversity based on the information theory.

The Simpson species evenness index (*D*) used in this study is given by Formula (2).

2 $$D=1-\sum _{i=1}^{s}{(\frac{{n}_{i}}{N})}^{2}$$

*D* profits into justification both the number of species and the equilibrium among them. The value of *D* falls within the interval [0…1] if there is only one species, *D* is zero. As the number of species increases – and their contribution to overall abundance is equalized – *D* approaches 1. The index offers a good approximation of diversity with a moderately small sample size, being less delicate near taxon fullness and netting the modification of the taxon abundance distribution^26^.

The Pielou species index (*J*) is calculated by the following formula (3).

3 $$J={H}/\mathrm{ln}\,S$$

The Margalef index (d_ma_) is calculated by: d_ma_ = (*S* − 1)/ln*N*.

*J* measures how evenly individuals are distributed among taxa in a community, d_ma_ reports the number of species corrected for sample abundance.

The Gleason index (d_gl_) is calculated by: d_gl_ = −*S*/ln*A*. *A* is its area where field survey conducted (in this study is a circle with radius of 0.8 meter).

### Hyperspectral data measurement and analysis

**Figure 2:**
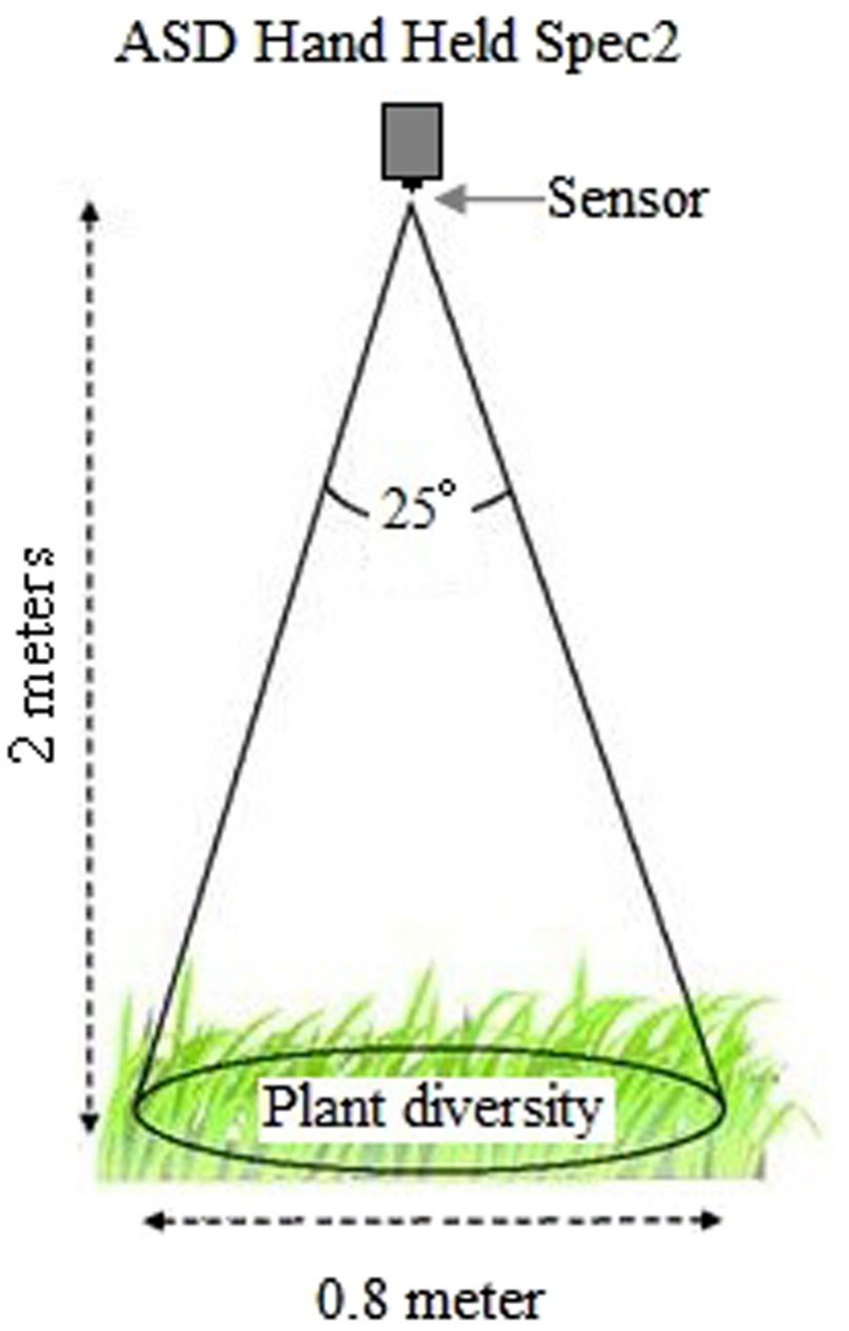
Illustration of the field measurement of spectral reflectance by ASD.The set up was adjusted until the sensor’s field-of-view (25°) was just within the circle.

Ground-based hyperspectral measurements were recorded concurrently for each subplot (a circle with diameter of 0.8 m) using a Hand–Held ASD portable FieldSpec 2 spectrometer (Analytical Spectral Devices Inc., USA). The spectrometer has a spectral range extending from 325 to 1075 nm, and a 1 nm bandwidth (www.asdi.com), weight in 1.2 kg. Measurements were taken during 10:00–15:00 (Beijing Time) in a sunny windless day in July, 2016. The surveyors dressed in dark and didn’t block the sun when measuring in order to minimize environmental reflections. Given the surveyed circle diameter of 80 cm, the canopy reflectance was measured by pointing the fibre optic with a field of view of 25 degrees in a nadir position, from about 200 cm above the centre of each surveyed circle (diameter 0.8 m), to ensure that only hyperspectral parameters within the surveyed circles were taken (Fig. 2). A white reference panel (spectralon) was used before each spectral measurement to convert spectral radiance into reflectance. Measuring followed the protocol used by e.g. Ramoelo *et al*. and Peng *et al*.^27,28^. Each quadrat (circle) was measured with 5 spectral replicates were taken and averaged to account for illumination differences and bi-directional reflectance effects^27^. In the raw data, the marginal ranges 325–380 nm and 1025–1075 nm from each spectrum were removed due to noise effects^29^. The quadrats were divided randomly into two datasets: 90 as the training dataset and the remaining 30 as the validation dataset for predicting plant diversity.

### Spectral indices

In this study 37 spectral vegetation indices^29,30^ were employed to estimate the plant diversity (Table 1). Based on the hypothesis of Gallardo-Cruz *et al*. (2012), the variation among primary reflectances at every band can be regarded as the proxy of species diversity^14^. Three indices were also established, as Hspec, Espec and VarH (Table 1) specifying the dissimilarity entropy and deviation among spectral reflectances, respectively, to estimate diversity of plant species. Other than these, the derivative values of reflectance are effective information to detect the variation among primary reflectances and have already been commonly applied in vegetation parameters analysis^31^. The first-order derivative values (FD) were calculated by finite difference approximation. FD = [*R*(λ + Δλ)-*R*λ]/Δλ, *R* is relative reflectance, λ is wavelength in nm, Δλ is the separation between adjacent bands.

**Table 1:** Hyperspectral indices used to estimate plant diversity.

Spectral index	Formula or definition
DVI	*R*_782_ − *R*_675_
NDVI	(*R*_782_ − *R*_675_)/(*R*_782_ + *R*_675_)
RVI	*R*_675_/*R*_782_
SAVI	((*R*_782_-*R*_675_)/(*R*_782_ + *R*_675_ + 0.2)) (1.2)
TSAVI	0.5(*R*_782_ − 0.5*R*_675_−0.2)/(0.5*R*_782_ + 0.5*R*_675_−0.1)
MSAVI	$2\ast {R}_{800}+1-{(2\ast {R}_{800}+1)}^{2}-8\ast {({R}_{800}-{R}_{670})}^{\frac{1}{2}}$
PVI	$({R}_{800}-0.2{R}_{670}-0.6)$/1.019
NDVI705	(*R*_750_ − *R*_705_)/(*R*_750_ + *R*_705_)
mNDVI705	(*R*_750_ − *R*_705_)/(*R*_750_ + *R*_705_ − 2 *R*_445_)
mSR705	(*R*_750_ − *R*_445_)/(*R*_705_ + *R*_445_)
REP	*R*_700_ + 40[(*R*_670_ + *R*_780_)/2 − *R*_700_]/(*R*_740_ − *R*_700_)
VOG1	*R*_740_/*R*_720_
VOG2	(*R*_734_ − *R*_747_)/(*R*_715_ + *R*_726_)
VOG3	(*R*_734_ − *R*_747_)/(*R*_715_ + *R*_720_)
PRI	(*R*_531_ − *R*_570_)/(*R*_531_ + *R*_570_)
OSAVI	(1 + 0.16)(*R*_800_ − *R*_670_)/(*R*_800_ + *R*_670_ + 0.16)
DVI	*R*_810_ − *R*_680_
GREEN-NDVI	(*R*_750_ − *R*_550_)/(*R*_750_ + *R*_550_)
FD_730_	The first derivative value at 730 nm
Db	The highest first derivative value between 490–530 nm
λb	The band at Db
Dy	The highest first derivative value between 550–580 nm
λy	The band at Dy
Dr	The highest first derivative value between 680–780 nm
Λr	The band at Dy
Rg	The highest reflectance value between 510–580 nm
Λg	The band at Rg
Ro	The smallest reflectance value between 640–700 nm
Λo	The band at Ro
SDb	The sum of first derivative values between 490–530 nm
SDy	The sum of first derivative values between 550–580 nm
SDr	The sum of first derivative values between 680–780 nm
Rg/Ro	Rg/Ro
(Rg-Ro)/(Rg + Ro)	(Rg − Ro)/(Rg + Ro)
SDr/SDb	SDr/SDb
SDr/SDy	SDr/SDy
(SDr-SDb)/(SDr + SDb)	(SDr-SDb)/(SDr + SDb)
Hspec*	$-\sum _{{\rm{i}}=1}^{{\rm{n}}}{{\rm{p}}}_{{\rm{i}}}{{\rm{lnp}}}_{{\rm{i}}}$
Espec*	$1-\sum _{{\rm{i}}=1}^{{\rm{n}}}{{{\rm{p}}}_{{\rm{i}}}}^{2}$
VarH**	$\frac{\sqrt{\frac{1}{{\rm{n}}}{\sum }_{{\rm{i}}=1}^{{\rm{n}}}{(R-\bar{R})}^{2}}}{\bar{R}}$

Note *pi: the ratio of *R* value at ith band to the sum *R* value; **$\bar{R}$: the mean value of *R*.

### Statistical analysis

The collected hyperspectral data were preprocessed by software ViewSpec Pro 6.0 (Analytical Spectral Devices Inc., USA), and exported into SPSS 22 (Statistical Package for the Social Sciences 22, Chicago, Illinois, USA) for correlation coefficients and stepwise regression analysis. The associations of FD at each band and the hyper spectral indices to plant species diversity was tested by correlation (Pearson correlation coefficients) and stepwise linear progression inquiry to define the most profound indices in assessing plant diversity. Stepwise regression requires that the number of training subplots be equal to or greater than the number of spectral indices, therefore, only the values of sensitive wavebands remained after the correlation coefficient analysis. RMSE (Root Mean Square Error), R^2^ and cAIC (corrected Akaike’s Information Criterion) of the linear regressions were taken into account for the selection of the maximum appropriate hyperspectral indices. The objective of cAIC is to select the best approximating model or set of models supported by the data. We selected the best fitting models using the following conditions: (1) the smallest cAIC and RMSE; (2) the largest r-square. The selected hyperspectral indices will be validated by field-survey data in another 30 quadrats.

## Results

### The spectral curves

**Figure 3:**
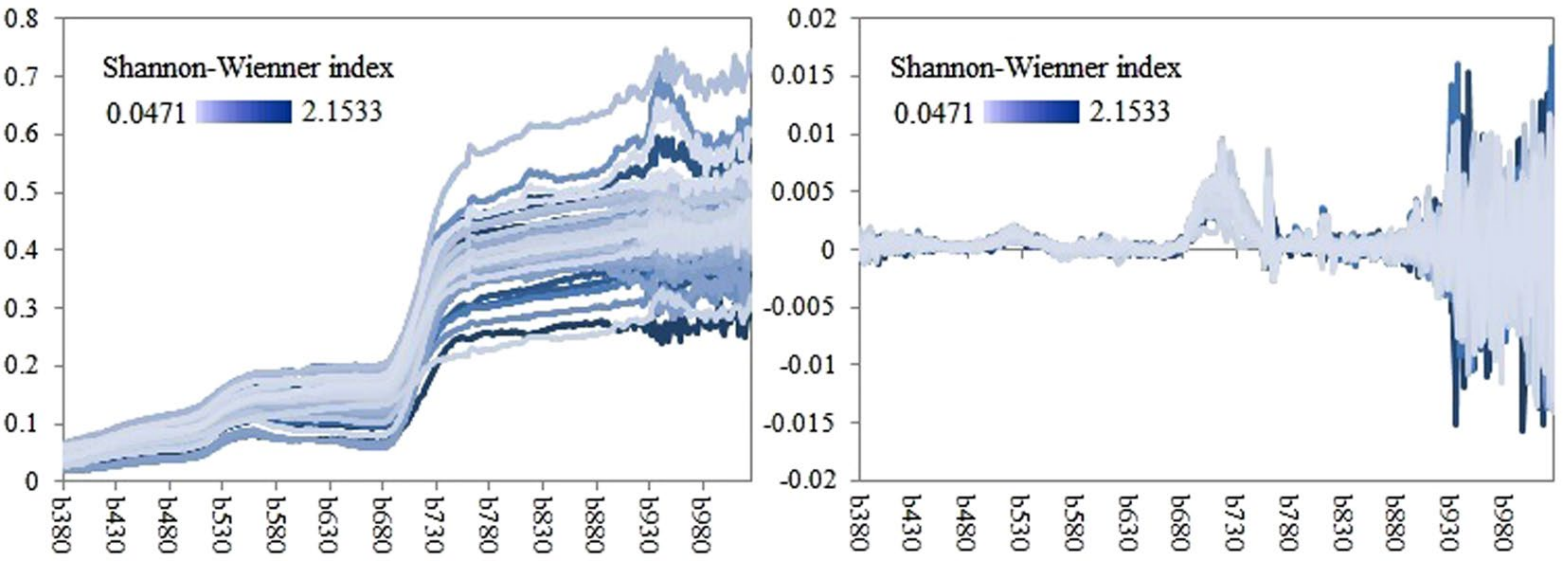
Mean reflectance spectra (left curves) and FD (right curves) from 90 quadrats collected in sandy grasslands in Hunshandak Sandland, Northern China.

We first examined the spectral curves (Fig. 3) of the quadrats in sandy grasslands in centre Hunshandak Sandland and estimated to what degree the spectral response distinguishes. It apparently the Shannon-wiener index was not closely associated to reflectance, as the black-blue curves (indicate a high Shannon-Wiener index) didn’t merge together (Fig. 3). The black-blue curves and light-blue curves (indicate a lower Shannon-Wiener index) were mixed, means that same color curves does not indicate same plant diversity. However, the FD curve obviously fluctuated more (either higher or lower than average) at some wavebands where indicated higher Shannon-Wiener index, representing a nearby connotation with plant diversity.

### The correlation between the first-order derivative values and plant diversity indices

Based on statistical analysis, plant diversity indices were non-significantly related to reflectance; however, plant diversity was closely related to FD values (Fig. 3). Examining the FD curves, we see that the sensitive bands were violet light (380–420 nm), blue light (450–470 nm), red light (616–655 nm) and NIR (764–1037 nm) (Supplemental Materials Fig. A). For violet light, the FD was significant positively related to the Shannon-Wiener index (0.41 < R < 0.55). Wavelengths of 412 and 417 nm were closely related to the Simpson and Pielou indices (0.46 < R < 0.48), while those of 421, 422, and 479 nm were closely related to the Marglef and Gleason indices (0.52 < |R| < 0.66). For red light, the FD primarily had a positive correlation with the Shannon-Wiener index (0.41 < R < 0.63). A wavelength of 623 nm was strongly negatively correlated with the Marglef and Gleason indices (R = −0.52), while those at 623, 654, and 655 nm were closely correlated with the Simpson and Pielou indices (0.43 < R < 0.71). For NIR light, the negative (764 to 769 nm) and positive (908 to 934 nm) correlations were observed between FD and Shannon-Wiener index and FD and the Simpson and Pielou indices, respectively. The Shannon-Wiener index was subtle to the first-order derivative value among the majority of bands within the ranges of violet light (380–420 nm), blue light (450–470 nm), red light (616–655 nm) and NIR (764–1037 nm). The Simpson and Pielou indices and Marglef and Gleason indices display comparable leanings to FD, respectively. Sensitive bands of 412, 417, 623, 639, 654, 655, 807, 923, 924, 933, 934 and 1026 nm were significantly positively related to the Shannon-Wiener, Simpson, and Pielou indices; those of 415, 421, 422, 479, 623, 819, and 1026 nm were closely related to the Marglef and Gleason indices.

Multiple linear stepwise regression analysis was also used to spot the associations between spectral first-derivative values and plant diversity indices. The determining coefficients (Table 2) and the relative fit indices (Supplemental Materials Table A) indicated that the regression models fit well, plant diversity indices can be nearly fully predicted by FD values (R^2^ ≅ 0.90 and low AICc). The Simpson, Shannon-Wiener, and Pielou indices were significantly associated with FD values at the bands of 654, 976, 790, 822, 852 and 970 nm, mainly belonged to red and NIR bands. The Margalef and Gleason indices were primarily determined by the FD values at the bands of 421, 911, 859, 800 and 441 nm.

**Table 2:** Regression equations for plant diversity based on the spectral first-derivative values in central Hunshandak Sandland, China.

Diversity indices	Regression equation	R^2^	Adjusted R^2^	RMSE
Simpson	Y = 2080.41FD_654_ − 60.515FD_976_ + 914.312FD_790_ + 504.106FD_822_ − 375.627FD_852_ + 0.247	0.894	0.863	0.011
Pielou	Y = 3003.342FD_654_ − 77.729FD_976_ + 69.338FD_966_ + 938.853FD_790_ + 1.087	0.889	0.864	0.043
Shannon-Wiener	Y = 321.434FD_654_ − 38.89FD_976_ + 31.274FD_966_ + 204.216FD_847_ + 122.714FD_853_ + 0.258	0.885	0.851	0.003
Margalef	Y = 4809.25FD_421_ − 268.FD_911_ − 431.53FD_859_ + 585.59FD_800_ + 5.292	0.831	0.795	0.102
Gleason	Y = 3718.52FD_421_ − 178.88FD_911_ − 225.22FD_859_ + 314.06FD_800_ + 51.163	0.843	0.809	3.031

### The correlation between hyperspectral indices and plant diversity indices

Supplemental Materials Fig. B grades the outcomes of the correlation investigation. All spectral indices have negative associations with the Shannon-Wiener index, with the exception of VOG2, VOG3, and PRI (0.41 < R < 0.66). The most related indices were (Rg-Ro)/(Rg + Ro) (R = −0.71), VOG1 (R = −0.67), Rg/Ro (R = −0.64), FD_730_ (R = −0.56). The Simpson and Pielou indices have the same tendency as Shannon-Wiener index. The Margalef and Gleason indices display negative relationship by means of all spectral indices. The closely associated spectral indices were Db (R = 0.75), FD730 (R = 0.74), Rg (R = 0.74), SDb (R = 0.74), SDr (R = 0.74), SDy (R = 0.74), Dy (R = 0.73~0.74), Dr (R = 0.73), Ro (R = 0.72), and (Rg-Ro)/(Rg + Ro) (R = 0.65~0.66). These correlation coefficients were higher than those in FD in Fig. 3, indicating that the hyperspectral indices based on multiple wavebands have greater potential for estimating plant diversity than does FD solely based on one waveband.

The results of regression analysis (Table 3, and Supplemental Materials Table B) indicate that (Rg-Ro)/(Rg + Ro), λb and Rg/Ro can accurately reflect the Simpson (R^2^ = 0.71) and Shannon-Wiener indices (R^2^ = 0.67). The Margalef and Gleason indices can be accurately simulated by Db (R^2^ = 0.55~0.56).

**Table 3:** Regression equations for plant diversity based on hyperspectral indices in central Hunshandak Sandland, Northern China.

Diversity indices	Regression equation	R^2^	Adjusted R^2^	RMSE
Simpson	Y = −4.873(Rg − Ro)/(Rg + Ro) + 0.509λb + 1.026Rg/Ro − 270.43	0.710	0.677	0.024
Pielou	Y = −0.699(Rg − Ro)/(Rg + Ro) + 0.244	0.403	0.382	0.013
Shannon-Wiener	Y = −9.697(Rg − Ro)/(Rg + Ro) + 0.974λb + 2.06Rg/Ro − 517.113	0.665	0.626	0.113
Margalef	Y = 2.984SDb + 21.595(Rg − Ro)/(Rg + Ro) − 9.176	0.554	0.539	0.455
Gleason	Y = 21.413SDb + 91.277(Rg − Ro)/(Rg + Ro) + 37.862	0.563	0.547	8.507

### Selection of hyperspectral indices and estimation of plant diversity

The optimal hyperspectral indices for estimating plant diversity were defined as the high R^2^ value, corresponding confident p-value, and low RMSE and cAIC. The first 10 best-performed hyperspectral indices were determined for each plant diversity index (Table 4).

**Table 4:** The best hyperspectral models identified for the estimation of plant diversity in central Hunshandak Sandland, Northern China.

Simpson	Pielou	Shannon-Wiener	Margalef	Gleason
y = 1585.5FD_654_ + 0.5873	y = 3486.4FD_654_ + 1.2107	y = 3486.4FD_654_ + 1.2107	y = 7385.2FD_421_ − 1.4331	y = 518107FD_421_ − 98.552
y = 930.19FD_639_ + 0.5832	y = 1871.5FD_639_ + 1.1802	y = 825.66FD_417_ + 0.6147	y = −2398.7FD_911_ + 5.1837	y = −168252FD_911_ + 365.61
y = 69.922FD_924_ + 0.4883	y = 1967.6FD_655_ + 1.241	y = 142.87FD_924_ + 0.9898	y = −965.31FD_991_ + 2.7834	y = −67543FD_991_ + 197.19
Y = 2080.41FD_654_ − 60.51 5FD_976_ + 914.312FD_790_+ 504.106FD_822_ − 375.627F D_852_ + 0.247	Y = 3003.342FD_654_ − 7 7.729FD_976_ + 69.338 FD_966_ + 938.853FD_79_ _0_ + 1.087	Y = 321.434FD_654_ − 38.89 FD_976_ + 31.274FD_966_ + 20 4.216FD _847_ + 122.714FD _853_ + 0.258	Y = 4809.25FD_421_ − 26 8.FD_911_−431.53FD_85_ _9_ + 585.59FD_800_ + 5.29 2	Y = 3718.52FD_421_ − 178.88FD_911_ − 225. 22FD_859_ + 314.06F D_800_ + 51.163
y = 0.7492VOG1−0.5344	y = 0.4532VOG1–0.401	y = 1.6 VOG1–1.1906	y = 65.007Rg − 6.0662	y = 467.95Rg − 43.776
y = −3.8796VOG2 + 0.2024	y = −2.3513VOG2 + 0.0443	y = −8.1799VOG2 + 0.3899	y = 78.699Db − 6.2515	y = 565.51Db − 44.993
y = −3.4672VOG3 + 0.2153	y = −2.1128VOG3 + 0.0514	y = −7.3178VOG3 + 0.4167	y = 2.1002SDb − 5.3066	y = 15.093SDb − 38.21
y = 0.2924Rg/Ro + 0.111	y = 0.1743Rg/Ro − 0.0076	y = 0.6362Rg/Ro + 0.174	y = 0.3552SDr − 7.5404	y = 2.5622SDr − 54.554
y = 0.9824(Rg − Ro)/(Rg + Ro) + 0.3973	y = 0.5662(Rg − Ro)/(Rg + Ro) + 0.1643	y = 2.1298(Rg − Ro)/(Rg + Ro) + 0.7972	y = 1.9632SDy − 5.8695	y = 14.123SDy − 42.32
y = −4.873(Rg − Ro)/(Rg + Ro) + 0.509λb + 1.026Rg/Ro − 270.43	y = −0.699(Rg − Ro)/(Rg + Ro) + 0.244	y = −9.697(Rg − Ro)/(Rg + Ro) + 0.974λb + 2.06Rg/Ro − 517.113	Y = 2.984SDb + 21.595(Rg − Ro)/(Rg + Ro) − 9.176	Y = 21.413SDb + 91.277(Rg − Ro)/(Rg + Ro) + 37.862

For the Simpson index, the sensitive indices were stepwise linear regression of FD (R^2^ = 0.90), followed by stepwise linear regression of spectral indices (R^2^ = 0.71), (Rg − Ro)/(Rg + Ro) (R^2^ = 0.531),and VOG1, VOG2, VOG3, Rg/Ro, FD_654_, FD_639_ and FD_924_. The seven most sensitive hyperspectral indices for the Pielou and Shannon-Wiener indices were the same as those of the Simpson index. The Margalef and Gleason indices shared the same mostly sensitive hyperspectral indices, such as the stepwise linear regression of FD (R^2^ = 0.90), Db, SDr, SDy, SDb, Rg, FD_421_, FD_911_, FD_991_, and the stepwise linear regression of spectral indices.

**Figure 4:**
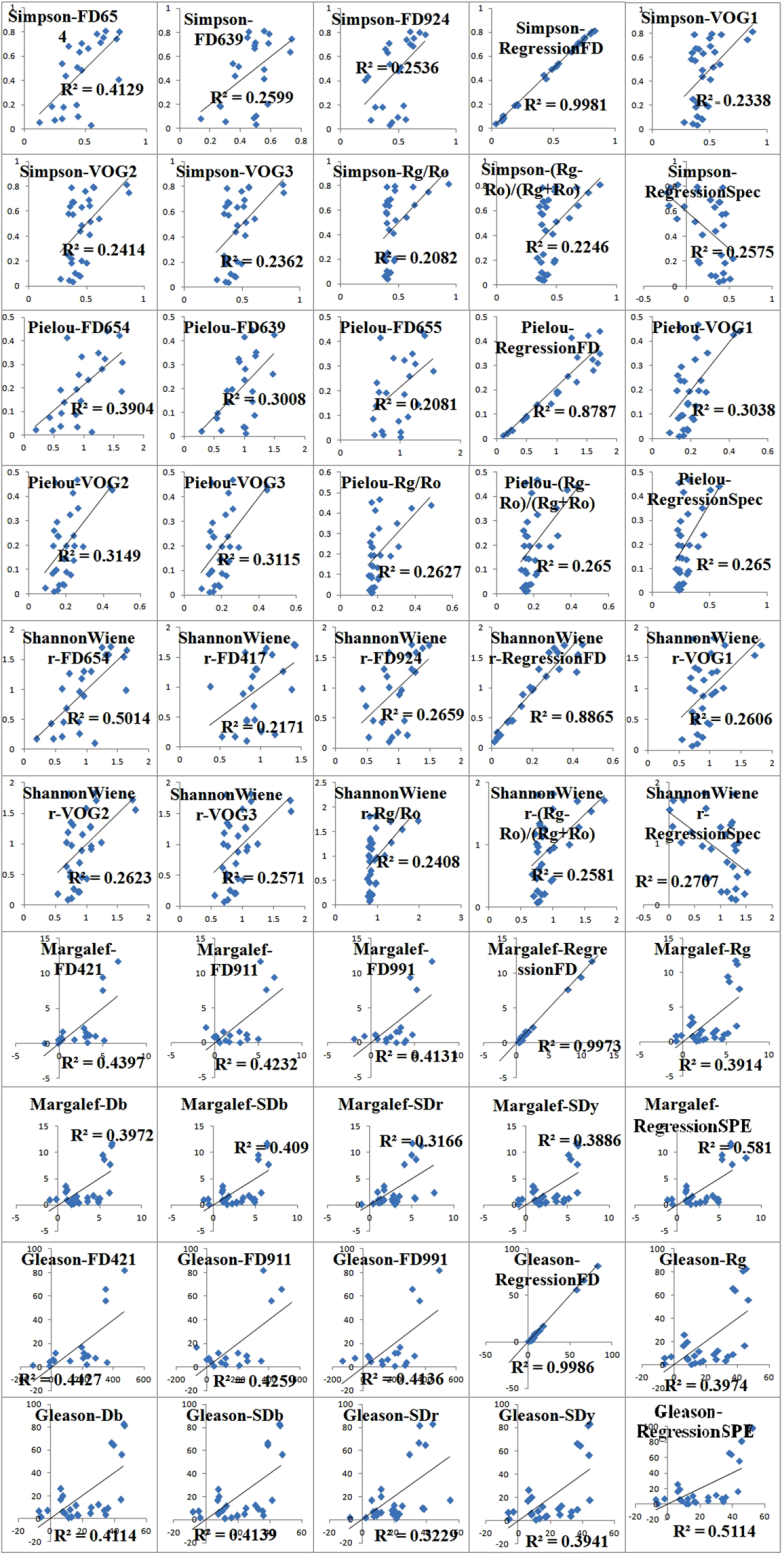
Linear regression of the field measured values (y-axis) and predicted values (x-axis) for plant species diversity indices in the central Hunshandak Sandland, Northern China. Predicted values were calculated based on the best hyperspectral indices in Table 4.

Based on the 10 identified best hyperspectral indices, five plant diversity indices were calculated using the hyperspectral dataset collected at another 30 quadrates (Table 4). The linear correlation between the estimated diversity by hyperspectral indices and the field-survey plant diversity was analyzed at quadrat level (Fig. 4). By comparison of R^2^, the best estimated hyperspectral indices (R^2^ > 0.5) for the Simpson index was the stepwise linear regression of FD (R^2^ = 0.83); for the Pielou index, the stepwise linear regression of FD (R^2^ = 0.87); for the Shannon-Wiener index, the stepwise linear regression of FD (R^2^ = 0.88) and FD_654_ (R^2^ = 0.5014); for both the Margalef and Gleason indices, the stepwise linear regression of FD (R^2^ = 0.82), and stepwise linear regression of hyperspectral indices (R^2^ = 0.51, R^2^ = 0.58, respectively).

## Discussion

The vegetation hyperspectral data delimited the spectral retorts from an assorted puddle of plant species, together with the durable gesture from the significant vital canopy of dominant species or young shoots with extra water content, rather than one species. Diverse with soil surface in the case of sparse vegetation in arid areas, all of these aspects potentially increase output noise^32^. Additionally, the acquired mixed reflectance of various plant species is influenced by the leaf cellular structure, leaf thickness, mesophyll structure, water content, and canopy architecture^32,33^. Grasses with small canopies typically have a lower ratio of “pure” inner canopy vs “mixed” outer canopy, making their detection more difficult unless if they are spectrally distinct from any background effects. We showed the wavebands assortment at the pre-processing phase. The sensitive wavebands selection was not only based on the consideration of correlation with plant diversity, but also the consideration of whether they were influenced by water and air absorption. Much of the information in a hyperspectral dataset may be redundant; nevertheless, important spectral information could be lost when only a small number of wavebands are used to predict species diversity indices^20^. Our selection of sensitive bands from 380–1025 nm has yielded desirable results than that based on empirical bands (Tables 2, 3, Fig. A1), or from a coarser scale (>4 m, r = 0.47~0.65)^20^, with moderately strong correlation coefficients ranging from 0.50 to 0.80 or even 0.90 in FD regression analyses.

The majority of plant species have their own unmatched reflectance curves^23^, and were especially different at the red and NIR bands^34^. The hyperspectral data collected from grass plots at a fine scale (0.8 m) reflected nearly all of the spectral information of every species fall in it, unlike the tree plots from which spectral data could not image the underlying shrub and herb layers^22^. Therefore, higher spectral variation is expected to be a good predictor for estimating species diversity at a fine scale. The FD is a significant indicator for the degree of deviation for reflectance across neighboring bands; it has been used to reduce the variation in spectral reflectance due to surface geometry, roughness, and the effects of water absorption feature on the spectrum^35^. Moreover, FD has the potential to eliminate background signals and overlapping spectral features^31^. In the current study, the FD models were greatly fruitful in approximating plant species diversity, particularly the linear stepwise regression model. This high estimation accuracy might be partially explained by the significant variation in the reflectance of sensitive bands. FD was also extensively used in numerous models for estimating vegetation parameters with a significantly advanced accuracy^31^ than other indices^36^. It is substance declaring that the selection of sensitive bands by linear stepwise regression can greatly improve the predictive performance of FD on plant diversity. Based on the stepwise linear regression of FD, the values of the Simpson, Pielou, Shannon-Wiener, Margalef and Gleason indices can be successfully predicted (R^2^ = 0.83; R^2^ = 0.87; R^2^ = 0.88; R^2^ = 0.81, R^2^ = 0.82). Thus, we strongly suggest that FD should be used as an independent variable for plant diversity estimation.

Among the 37 hyperspectral indices in this study, more indices were significantly correlated with the Marglef and Gleason indices, than with the Simpson, Pielou or Shannon-Wiener indices. This difference might be due to the effect of dominant species on reflectance in complex grass communities, i.e., the strong signal from canopy species might not scale with the species abundance and richness^22^. The Margalef and Pielou indices are strongly influenced by dominant species^26^, this is one plausible reason that these indices can be well-simulated by more hyperspectral indices than other diversity indices. The significant negative correlations between reflectance and diversity in the NIR spectral bands (Supplemental Materials Fig. A) indicated that the species diversity increased as the above-ground coverage decreased; this result is consistent with those of other studies^20^. We found that, in some subplots where the diversity was higher while the coverage was lower, small herb species with little coverage likely contributed to the Shannon-Wiener and Simpson diversities. As for the Margalef and Gleason indices, the negative correlation with spectral indices might be attributed to the method of index calculation, which was influenced by the plot area and total number of species.

The calculation of species diversity over and done with the spectral indices by numerous combinations on NIR wavebands has been extensively documented^32,37^. Other than NIR bands, our study has demonstrated that the visible wavebands (blue, yellow, and red) might also contain important information pertaining to plant species diversity, especially for the Pielou and Gleason indices (Rg/Ro, Db). The positive correlation observed between the regression on (Rg-Ro)/(Rg + Ro), λb indices and the Shannon-Wiener and Simpson indices (Supplemental Materials Fig. B) which might be due to the high variation in the chlorophyll absorption bands of the spectrum (blue and red)^19,38^.

Conflicting to our anticipation, the entropy (Hspec, Espec) of spectral evidence was not suggestively linked to plant diversity and the deviation of spectral difference (VarH) displayed a significant positive relationship with plant diversity (Supplemental Materials Fig. B). Similar results have been testified by another case study at a scale of 4 m^20^. A study on North American plant species richness described that the spectral diversity clarified a slight quantity of the dissimilarity in plant diversity, while the spatial extent of the sampling unit explained a large amount of the variation^3^. The failure to detect a significant relationship between spectral variation and species diversity in our study might be due to the entropy index being calculated by all selected bands through 380–1025 nm rather than red, NIR bands separately^39^. Thus, the spectral entropy primarily reflected the information of entire objects (vegetation and non-vegetation) within subplots, rather than the variation among various plant species.

In the present study, the mosaic grass canopy of the quadrats almost includes all shoots of every species within it, unlike the tree canopy which cannot image the spectral characteristics of the underlying shrub and herb layers^22^. This grantee the diverse hyperspectral parameters can provide the mostly information of all grass species^29^. Some case studies demonstrated that the best spectral indices to explain variation in plant species were conducted at lower levels of biomass^40^. Concerning the sparse vegetation on the sandy dunes in Hunshandak Sandland, the high accuracy in estimating plant diversity might also be attributed to the fine scale and lower biomass from which hyperspectral data were obtained.

Through background noise elimination, sensitive bands selection, first-order derivative value calculation at the pre-processing stage, and stepwise regression on sensitive bands and spectral vegetation indices, plant species diversity of grasslands at a fine scale can be predicted accurately. Compared to airborne hyperspectral imagery, the hyperspectral data gained by hand-held portable parameters have the advantages of low labor cost and high spatial resolution, and they are less influenced by atmosphere layer and background environment; therefore they might be a better option for quick estimation of plant diversity. These characteristics are important when carrying out repeat monitoring on fine-habitat species diversity over large areas, especially for grasslands since they cover nearly a third of the continents on earth^41^.

Except for plant diversity estimation, the methodology used in the present study can help in recovering estimates made through remote sensing data for other ecological applications^41,42^. Ecological condition evaluations, such as riparian condition^43^, vegetation eco-restoration^44^ and forest cover mapping^45^, all of which are variables derived from satellite or airborne imageries at a grain of 30 m or coarser, might be improved by hyperspectral data and stepwise linear regression on narrower sensitive wavebands. To improve airborne imagery, hyperspectral data tends to average a very large number of fine-plots to provide reflectance at a coarser scale^3,23,39^. The combination of hyperspectral indices and satellite/airborne imageries through scale conversion can extend the scope of using remote sensing. Directing on desert undergrowth, grassland, and pasture habitats, future efforts will explore the relationships of spatial and spectral resolutions on the performance of each hyperspectral model. Such research will help us to better recognize the trustworthiness of hyperspectral models and the degree of the scope of its application.

## Conclusion

Based on a correlation analysis between numerous plant diversity indices and reflectance characteristics, the capability of hyperspectral reflectances to estimate plant diversity was evaluated through background noise elimination, sensitive bands selection, first-order derivative value calculation, and subsequent stepwise regression. Based on these processes, plant diversity at a fine scale was accurately predicted by hyperspectral indices.

This research reinforces the growth of approaches for estimating plant diversity based on hyperspectral data. Future work will encompass results from multiple hyperspectral sources other than ASD, as LiDAR and aerial multispectral data, to make accurate comparisons when estimating plant diversity and other ecological observes. This will put onward more specific and public execution by hyperspectral data.

## Electronic supplementary material

Supplementary information
