# Supplementary material for: Assessment of plant species diversity based on hyperspectral indices at a fine scale
Source: Sci Rep. 2018 Mar 19;8:4776. doi: 10.1038/s41598-018-23136-5 (PMC5859024; doi:10.1038/s41598-018-23136-5)
Supplement: Supplementary file 1 — Supplementary information [file 41598_2018_23136_MOESM1_ESM.pdf]

Supplement Materials for manuscript SREP- 17-33749

Assessment of plant species diversity based on hyperspectral indices at a fine scale

Yu Peng*, Min Fan, Jingyi Song, Tiantian Cui, Rui Li

College of Life & Environmental Sciences, Minzu University of China, Haidian District, Beijing 100081, China. Correspondence and requests for materials should be addressed to Y. Peng (email: yuu.peng@muc.edu.cn)

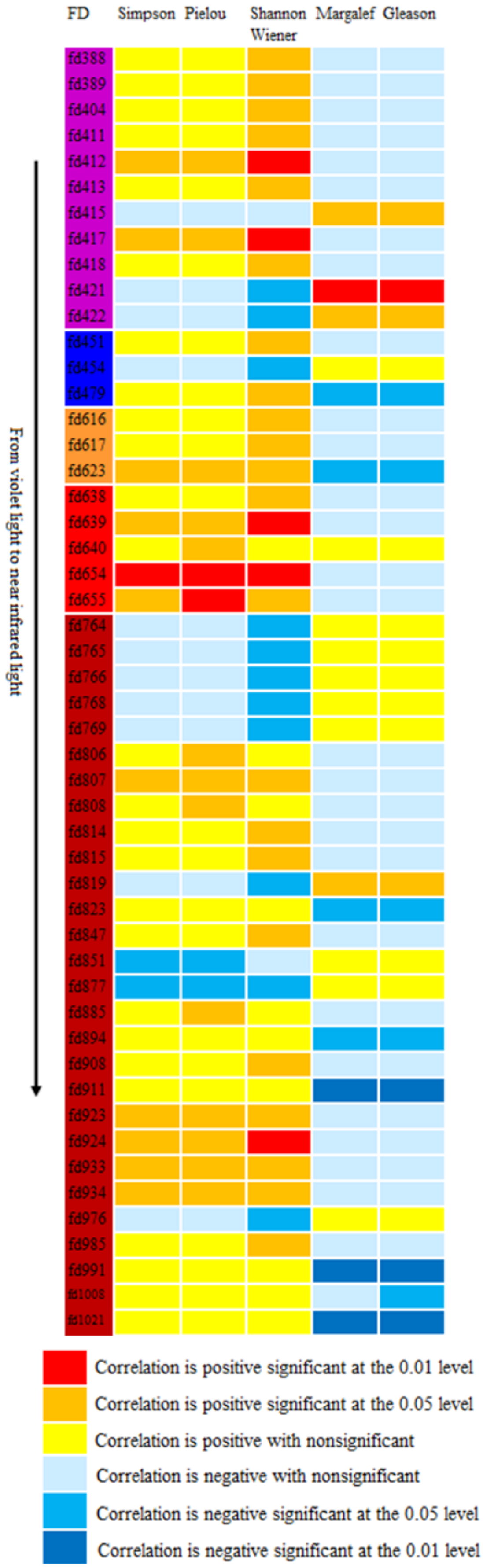
FD
Simpson
Pielou
Shannon Wiener
Margalef
Gleason
From violet light to near infrared light
fd388
fd389
fd404
fd411
fd412
fd413
fd415
fd417
fd418
fd421
fd422
fd451
fd454
fd479
fd616
fd617
fd623
fd638
fd639
fd640
fd654
fd655
fd764
fd765
fd766
fd768
fd769
fd806
fd807
fd808
fd814
fd815
fd819
fd823
fd847
fd851
fd877
fd885
fd894
fd908
fd911
fd923
fd924
fd933
fd934
fd976
fd985
fd991
fd1008
fd1021
Correlation is positive significant at the 0.01 level
Correlation is positive significant at the 0.05 level
Correlation is positive with nonsignificant
Correlation is negative with nonsignificant
Correlation is negative significant at the 0.05 level
Correlation is negative significant at the 0.01 level

Figure A. Correlation coefficients between the spectral first-order derivative values and field measured plant diversity indices in central Hunshandak Sandland, China (n=90). Different color indicated various Pearson's correlations.

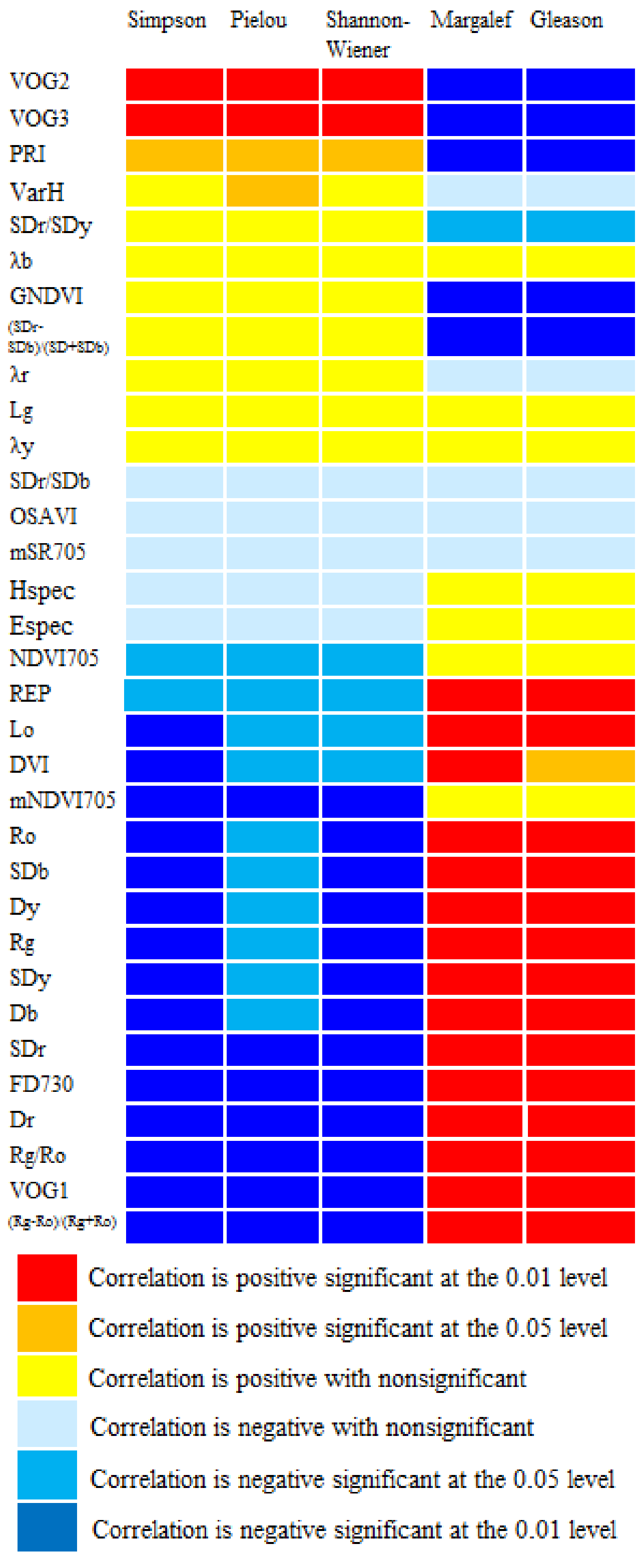

Figure B. Correlation coefficients between hyperspectral indices and field measured plant species diversity indices in central Hunshandak Sandland, China (n=90). Different color indicated various Pearson's correlations.

Table A The statistical parameters of FD based regression equations on plant diversity indices

Diversity indices	Regression equation	F	p	cAIC
Simpson	$Y=2080.41FD_{654}-60.515FD_{976}+914.312FD_{790}+504.106FD_{822}-375.627FD_{852}+0.247$	28.724	0.001	-5.037
Pielou	$Y=3003.342FD_{654}-77.729FD_{976}+69.338FD_{966}+938.853FD_{790}+1.087$	35.995	0.001	-3.618
Shannon-Wiener	$Y=321.434FD_{654}-38.89FD_{976}+31.274FD_{966}+204.216FD_{847}+122.714FD_{853}+0.258$	26.208	0.001	-6.350
Margalef	$Y=4809.25FD_{421}-268.FD_{911}-431.53FD_{859}+585.59FD_{800}+5.292$	40.483	0.003	5.971
Gleason	$Y=3718.52FD_{421}-178.88FD_{911}-225.22FD_{859}+314.06FD_{800}+51.163$	42.515	0.003	5.213

Table B The statistical parameters of FD based regression equations on plant diversity indices

Diversity indices	Regression equation	F	p	cAIC
Simpson	Y=-4.873(Rg-Ro)/(Rg+Ro)+0.509λb+1.026Rg/Ro-270.43	21.230	0.001	-3.883
Pielou	Y= -0.699(Rg-Ro)/(Rg+Ro)+0.244	18.927	0.001	-4.475
Shannon-Wiener	Y=-9.697(Rg-Ro)/(Rg+Ro)+0.974λb+2.06Rg/Ro-517.113	17.213	0.001	-2.342
Margalef	Y = 2.984SDb +21.595(Rg-Ro)/(Rg+Ro)- 9.176	34.843	0.005	9.792
Gleason	Y=21.413SDb+91.277(Rg-Ro)/(Rg+Ro)+37.862	36.085	0.004	9.058
